# Social Withdrawal, Eating Behaviors, and Daytime Sleepiness in Adolescents with Obesity: A Biopsychosocial Case–Control Study

**DOI:** 10.3390/children13040550

**Published:** 2026-04-15

**Authors:** Pınar Algedik, Sibel Ergin Şahin, Orhan Kocaman, Ezgi Özkan, Heves Kırmızıbekmez

**Affiliations:** 1 Department of Psychiatry, Faculty of Medicine, Haliç University, 34060 Istanbul, Turkey; 2 Department of Pediatric Endocrinology, Ümraniye Training and Research Hospital, 34764 Istanbul, Turkeyheves.kirmizibekmez@sbu.edu.tr (H.K.); 3 Department of Child and Adolescent Psychiatry, Faculty of Medicine, Alanya Alaaddin Keykubat University, 07450 Alanya, Turkey; 4 Department of Psychology, Istanbul Kültür University, 31456 Istanbul, Turkey

**Keywords:** adolescent obesity, social withdrawal, Hikikomori risk, emotional eating, uncontrolled eating, sleep disturbances, daytime sleepiness, self-regulation, biopsychosocial model, case–control study

## Abstract

**Highlights:**

**What are the main findings?**

• Adolescents with obesity demonstrate significantly higher levels of social withdrawal, particularly in anthropophobia, agoraphobia, lethargy, and depressive mood dimensions.
• Obesity is moderately associated with uncontrolled and emotional eating behaviors, and daytime sleepiness is closely associated with social withdrawal and dysregulated eating rather than the BMI z-scores.

**What are the implications of the main findings?**

• Adolescent obesity should be approached through a biopsychosocial framework that integrates social withdrawal, sleep-related vulnerabilities, and eating behaviors.
• Incorporating emotion regulation, sleep hygiene, and social engagement strategies into clinical practice may improve the identification and management of adolescents with obesity who require broader psychosocial support.

**Abstract:**

Background/Objectives: Adolescent obesity is increasingly recognized as a multidimensional condition involving psychosocial and behavioral vulnerabilities beyond metabolic risk. Although social withdrawal, dysregulated eating behaviors, and sleep disturbances have each been associated with obesity, integrative studies examining these domains concurrently remain limited. This study aimed to comparatively evaluate social withdrawal characteristics, eating behavior patterns, and daytime sleepiness in adolescents with obesity and normal-weight peers. Methods: This cross-sectional case–control study included 209 adolescents aged 14–18 years (100 with obesity; 109 normal-weight controls). Social withdrawal was assessed using the Hikikomori Risk Inventory (HRI-24), eating behaviors with the Children’s Three-Factor Eating Questionnaire (CTFEQ-17), and daytime sleepiness with the Pediatric Daytime Sleepiness Scale (PDSS). The BMI z-scores were calculated according to the CDC growth charts. Group comparisons and correlation analyses were performed. Results: Adolescents with obesity demonstrated significantly higher total social withdrawal scores and higher anthropophobia, agoraphobia, lethargy, and depressive mood subscale scores compared with the controls (all *p* < 0.01). Uncontrolled eating and emotional eating were also significantly higher in the obesity group (both *p* < 0.001), whereas cognitive restraint did not differ between groups (*p* > 0.05). Daytime sleepiness scores were higher in adolescents with obesity (*p* < 0.01). The BMI z-scores were positively correlated with social withdrawal dimensions and dysregulated eating behaviors (r = 0.15–0.30, *p* < 0.05) but not with daytime sleepiness. In contrast, daytime sleepiness was moderately associated with social withdrawal and uncontrolled/emotional eating (all *p* < 0.001). Conclusions: Adolescent obesity is associated not only with maladaptive eating behaviors but also with broader psychosocial vulnerabilities, including social withdrawal tendencies and sleep-related difficulties. These findings support a biopsychosocial conceptualization of adolescent obesity and underscore the importance of multidimensional intervention approaches targeting emotional regulation, sleep hygiene, and social functioning alongside weight management strategies.

## 1. Introduction

Adolescent obesity has become a major global public health concern, with prevalence rates continuing to rise in developed and developing countries. Recent epidemiological evidence indicates that the prevalence of obesity among children and adolescents has increased markedly over recent decades and now affects millions of young people worldwide [1,2]. Recent studies from Türkiye indicate a similar increasing trend in the prevalence of obesity among children and adolescents [3]. Excess body weight is associated with cardiometabolic disorders, type 2 diabetes, and an increased risk of long-term cardiovascular morbidity in adulthood [4]. However, beyond its well-established metabolic consequences, obesity during adolescence should also be considered a significant developmental challenge. Adolescence is a sensitive period characterized by rapid biological, cognitive, and psychosocial changes, during which body image, peer acceptance, emotional regulation, and identity formation become central developmental tasks. During this stage, excess weight has been associated not only with physical health complications but also with depressive symptoms and heightened psychological vulnerability [5]. These findings suggest that adolescent obesity is not merely a condition of excessive adiposity but rather a multidimensional health issue shaped by complex biopsychosocial processes.

Obesity during adolescence is associated not only with physical health risks but also with significant difficulties in social and emotional functioning. Meta-analytic findings indicate that children and adolescents with overweight and obesity are more frequently exposed to peer bullying and victimization compared with their normal-weight peers [6,7]. Longitudinal studies have demonstrated that experiences of weight-based teasing and bullying are associated with lower self-esteem, poorer body image, and increased depressive symptoms over time [8]. In addition, cross-sectional evidence suggests that adolescents exposed to weight-based teasing exhibit poorer psychological well-being, lower physical self-concept, reduced physical activity self-efficacy, and less favorable health-related fitness profiles [9]. Moreover, large-scale cohort data show that diagnoses of anxiety and depression are more prevalent among children and adolescents with obesity, even after controlling for socioeconomic and familial factors [10]. These findings suggest that adolescent obesity is not merely a metabolic condition but may also constitute part of a broader pattern of psychosocial vulnerability characterized by social exclusion, difficulties in peer relationships, and heightened internalizing symptoms. However, in the existing literature, the relationship between obesity and structured measures of social withdrawal has received comparatively less attention than internalizing symptoms such as depression and anxiety.

Adolescence-related obesity is closely linked to self-regulation processes underlying eating behavior. Contemporary neurobehavioral models conceptualize adolescent obesity not merely regarding increased energy intake but also in relation to heightened reward system sensitivity, weaknesses in executive functioning, and limitations in inhibitory control [11,12]. Adolescence represents a developmental period characterized by the relative hyperactivity of the dopaminergic reward system, while the prefrontal cortex-based inhibition and self-control mechanisms are not yet fully mature; this imbalance may lead to increased sensitivity to high-energy foods and a stronger propensity for impulsive eating behaviors [13]. Longitudinal and population-based studies indicate that eating in response to stress and stress-related eating behaviors are associated with increased BMI and an elevated risk of obesity [14]. Difficulties in emotion regulation have been shown to be strongly associated with eating disorder symptoms and particularly with impulsivity and emotional control problems linked to overeating patterns [15]. Using eating as a strategy to regulate negative affect may provide short-term relief; however, it may create a cycle that complicates long-term weight regulation. Furthermore, attempts at cognitive restraint are not always protective; a higher BMI has been found to predict higher engagement in restrained eating among adolescents, yet such efforts do not consistently prevent weight gain over time [16]. Taken together, these findings suggest that adolescent obesity should not be understood solely as a quantitative problem of excessive intake but rather as a complex self-regulation difficulty shaped by the interaction of reward processing, executive functioning, and emotion regulation systems. This framework theoretically supports the assumption that social withdrawal and negative affect may increase tendencies toward uncontrolled and emotional eating.

Another important regulatory factor associated with obesity during adolescence is sleep and circadian rhythm characteristics. Adolescence is a developmental period biologically characterized by a pronounced delay in sleep phase, a shift in melatonin secretion to later hours, and the widespread occurrence of chronic sleep restriction. Social and academic demands accompanying these physiological changes may lead to insufficient and irregular sleep patterns in adolescents. Meta-analytic and prospective cohort findings indicate that short sleep duration and irregular sleep timing are associated with an increased risk of obesity in children and adolescents [17,18]. Experimental studies further demonstrate that sleep restriction may lead to increased hedonic eating tendencies and heightened neural responsivity in reward-related brain regions to high-calorie food cues [19,20]. In addition, insufficient sleep weakens executive functioning and inhibitory control, which may particularly increase impulsive and emotional eating behaviors. Associations have also been reported between late chronotype and social jetlag with increased energy intake, night snacking, and higher BMI in adolescents [21,22]. Sleep irregularity is likewise associated with emotion regulation difficulties and internalizing symptoms, suggesting that sleep may constitute a potential biopsychosocial bridge linking social withdrawal and uncontrolled eating behaviors. Within this framework, sleep should be considered not merely a co-occurring variable in adolescent obesity but a central regulatory factor that may influence self-regulation processes and psychosocial functioning.

The existing literature has largely separately examined psychosocial difficulties, eating behavior patterns, and sleep disturbances in adolescent obesity. While the associations between obesity and peer bullying, internalizing symptoms, and emotional eating are well documented, studies that systematically address structured measures of social withdrawal (e.g., multidimensional risk inventories) within the context of obesity remain relatively limited. Similarly, although the relationship between sleep characteristics and obesity has been widely investigated, integrative models that comparatively evaluate social withdrawal, eating behavior patterns, and daytime sleepiness within the same sample are not sufficiently represented. Therefore, to better understand the biopsychosocial nature of adolescent obesity, there is a need for integrated studies that examine self-regulation processes, social functioning, and sleep regulation within a unified analytical framework.

This study conceptualizes adolescent obesity not merely as an imbalance in energy intake and expenditure but as a manifestation of multidimensional difficulties in self-regulation processes. Within this framework, social withdrawal tendencies, emotional and uncontrolled eating behaviors, and disturbances in sleep patterns are assumed to interact on a shared self-regulatory basis. During adolescence, the developmental imbalance between heightened reward system sensitivity and still-maturing executive functions may influence eating behaviors and social functioning, while sleep dysregulation may further exacerbate these processes. Sleep–wake regulation processes, including circadian rhythm alignment and sleep timing, may play a central role in modulating emotional regulation and reward-driven behaviors, thereby linking daytime sleepiness with social withdrawal and dysregulated eating patterns. Accordingly, the present study aims to simultaneously evaluate and integrate social withdrawal, eating behaviors, and daytime sleepiness within an integrated biopsychosocial framework.

In line with this framework, the present study was designed to address the following research questions and corresponding hypotheses:

(1) Are there significant differences between adolescents with obesity and their normal-weight peers regarding social withdrawal, eating behaviors, and daytime sleepiness? We hypothesize that adolescents with obesity will demonstrate higher levels across these domains.
(2) Are BMI z-scores associated with social withdrawal, eating behaviors, and daytime sleepiness? Positive associations between BMI z-scores and these variables are expected.
(3) Does daytime sleepiness emerge as an independent factor associated with social withdrawal and eating behaviors? We hypothesize that daytime sleepiness will be independently associated with social withdrawal and dysregulated eating behaviors.

This study aims to contribute to the literature by employing a case–control design to simultaneously examine social withdrawal, eating behaviors, and daytime sleepiness within the same sample using validated multidimensional assessment tools.

## 2. Materials and Methods

### 2.1. Study Design and Participants

This cross-sectional case–control study was conducted at the pediatric endocrinology outpatient unit between November 2024 and September 2025.

The final study sample consisted of 209 adolescents aged 14–18 years, including 100 adolescents with obesity and 109 age-matched normal-weight controls. Adolescents in the obesity group were recruited consecutively from outpatient clinic admissions, while the control group comprised age-matched adolescents without obesity who were recruited from the same clinical setting and did not meet any of the predefined exclusion criteria, ensuring that they were free from conditions that could affect body weight, eating behavior, or sleep patterns.

### 2.2. Inclusion and Exclusion Criteria

Adolescents aged 14–18 years who provided written informed consent from themselves and their parents or legal guardians, who were able to complete all assessment tools independently, and who had complete and valid questionnaire data were included in the study.

The obesity group consisted of adolescents with a body mass index (BMI) at or above the 95th percentile for age and sex, as defined according to the 2000 CDC Growth Charts.

The body mass index (BMI) was calculated, and age- and sex-specific BMI z-scores were determined using the 2000 CDC Growth Charts as the reference. Obesity was defined as a BMI at or above the 95th percentile according to the CDC criteria [23].

The control group included adolescents with a BMI between the 5th and 85th percentiles, indicating normal weight status, calculated based on the objectively measured height and weight values obtained during clinical evaluation.

BMI percentiles and BMI z-scores were calculated using age- and sex-specific reference values.

The exclusion criteria were as follows:

Presence of a chronic medical condition that could affect body weight, eating behavior, or sleep patterns;

History of neurodevelopmental disorders (e.g., autism spectrum disorder, intellectual disability);

Use of psychotropic or systemic medications known to influence appetite, sleep, or body weight;

A known diagnosis of psychiatric disorders, including depression and anxiety, based on clinical evaluation and medical records;

Incomplete, inconsistent, or invalid questionnaire data;

Failure to meet the predefined BMI criteria for either group.

Participants were also excluded if more than 10% of the items on any of the administered questionnaires were missing. Inconsistent or invalid responses were defined as patterns indicating random or non-engaged responding (e.g., identical responses across all items or logically contradictory answers), and such cases were excluded from the analyses.

### 2.3. Sociodemographic and Clinical Variables

Data on the participants’ age, sex, and anthropometric measurements (weight, height, body mass index [BMI], and BMI z-scores) were collected using a structured sociodemographic data form developed by the researchers. In addition, information regarding eating behaviors, sleep-related characteristics, and daily activity levels was obtained through this form.

Family-related variables included the parental age, education level, monthly household income, and number of siblings, all of which were recorded based on parental self-report.

Anthropometric measurements were obtained during the clinical evaluation by trained healthcare personnel, with participants wearing light clothing and no shoes, using standardized measurement procedures.

### 2.4. Assessment Instruments

#### 2.4.1. Hikikomori Risk Inventory (HRI)

The Hikikomori Risk Inventory (HRI) was used to assess social withdrawal and associated psychological characteristics in adolescents [24]. The inventory consists of 24 items and provides a total score as well as five subscale scores, including social avoidance (anthropophobia), agoraphobia, paranoia, lethargy, and depressive mood.

Items are rated on a Likert-type scale (1 = strongly disagree to 5 = strongly agree), with higher scores indicating higher levels of social withdrawal and related psychological difficulties. The total and subscale scores are calculated by summing the relevant items. The validity and reliability of the Turkish version of the HRI-24 have been established in a validation study [25], and the construct validity of the scale was further supported by the CFA results obtained in the present study. For the HRI-24, Cronbach’s alpha was 0.91 for the total score and ranged between 0.71 and 0.82 for its subscales.

#### 2.4.2. Three-Factor Eating Questionnaire (CTEFQ)

Eating behaviors were assessed using the 17-item version of the Children’s Three-Factor Eating Questionnaire (CTFEQ-17) [26], which evaluates three core dimensions of eating behavior:

Cognitive restraint;

Uncontrolled eating;

Emotional eating.

Items are rated on a four-point Likert scale, and higher scores on the uncontrolled eating and emotional eating subscales reflect higher impulsive and affect-driven eating tendencies. The subscale scores are calculated by summing item responses.

For the CTFEQ-17, items 1–16 were reverse-coded where necessary to ensure consistency with the original scoring procedure, as recommended by Demir et al. [27]. Item 17 was scored in accordance with the original scale instructions. Items were coded in accordance with the original scoring procedure recommended for the Turkish version of the scale. For the CTFEQ-17, Cronbach’s alpha values for the subscales ranged between 0.76 and 0.92.

#### 2.4.3. Pediatric Daytime Sleepiness Scale (PDSS)

Daytime sleepiness was evaluated using the Pediatric Daytime Sleepiness Scale (PDSS), a self-report instrument consisting of eight items designed to assess daytime sleepiness in children and adolescents [28].

Items are rated on a Likert-type scale, yielding a total score, with higher scores indicating higher levels of daytime sleepiness. The total score is calculated by summing all item responses. Item 3 was reverse-coded prior to analysis, in line with the original scale instructions. For the PDSS, the Cronbach’s alpha coefficient for the total score was 0.80.

### 2.5. Statistical Analysis

Statistical analysis of the data was performed using Jamovi software (version 2.7.17, The Jamovi Project). Descriptive statistics for the sociodemographic characteristics and anthropometric measurements of the participants were presented as the mean and standard deviation (Mean ± SD) for continuous variables and as the frequency (*n*) and percentage (%) for categorical variables. The normality of the data was assessed using the Shapiro–Wilk test, along with the evaluation of skewness and kurtosis values. All continuous variables had skewness and kurtosis values within the acceptable range of −1.5 to +1.5, and therefore, the assumption of normality was considered to be met. Parametric tests were employed accordingly. Confirmatory factor analysis (CFA) was conducted to test the construct validity of the scales. The sample size (*n* = 209) was considered adequate for CFA based on the commonly accepted recommendations for sample size in structural equation modeling. The model fit was evaluated based on the Chi-square (χ^2^), Degrees of Freedom (df), Comparative Fit Index (CFI), Tucker–Lewis Index (TLI), and Root Mean Square Error of Approximation (RMSEA) goodness-of-fit indices. Measurement invariance analyses, including configural, metric, and scalar stages, were performed to determine whether the scales were structurally equivalent across the obesity and control groups. The internal consistency reliability coefficients of the scales were calculated using Cronbach’s alpha (α). The Independent Samples t-test was used to compare continuous variables between the groups (Obesity and Control). Cohen’s d coefficient was calculated to determine the effect size of the differences. Effect sizes were interpreted according to Cohen’s criteria, where d = 0.20 indicates a small effect, 0.50 a medium effect, and 0.80 a large effect. These effect size estimates were also considered to provide an indication of the clinical relevance of the observed group differences. For the comparison of categorical variables between groups, the Pearson Chi-square (χ^2^) test was applied. The Pearson correlation coefficient (r) was used to examine the relationships between the body mass index (BMI) z-scores and scale scores. Statistical significance was defined as a *p*-value of less than 0.05 for all analyses. To control for Type I error inflation due to multiple comparisons, the Benjamini–Hochberg procedure was applied.

## 3. Results

The study sample consisted of 209 adolescents aged between 14 and 18 years, with 100 adolescents in the obesity group and 109 adolescents in the normal-weight control group. The mean age of the overall sample was 15.0 ± 1.75 years, with 177 (84.7%) female participants and 32 (15.3%) male. The descriptive data regarding anthropometric measurements (body weight, height, body mass index [BMI], and BMI z-scores), as well as parental characteristics (age, BMI, and educational levels of mothers and fathers) and household income levels, are presented in Table 1.

The distribution of weight change categories differed between the groups, with a higher proportion of participants reporting weight gain in the obesity group (*p* = 0.032). No statistically significant difference was observed between the groups regarding the mean number of main meals per day (*p* = 0.295). In contrast, the mean number of daily snacks was higher in the obesity group compared with the control group (*p* = 0.005). Meal skipping behavior did not differ significantly between the groups (*p* = 0.054). The night eating habits differed between the groups, with a higher proportion of adolescents reporting night eating in the obesity group (*p* = 0.031). The daily time spent indoors was higher in the obesity group, and the proportion of participants reporting having social relations was lower in the obesity group (both *p* < 0.001) (Table 2).

The HRI-24 total score differed between the obesity and control groups (*p* = 0.001). When the HRI-24 subscales were examined, the anthropophobia (*p* < 0.001), agoraphobia (*p* < 0.001), lethargy (*p* < 0.001), and depressive mood (*p* = 0.009) scores differed between the groups, whereas no difference was observed for the paranoia subscale (*p* = 0.805). Regarding the CTFEQ-17 subscales, the uncontrolled eating (*p* < 0.001) and emotional eating (*p* < 0.001) scores differed between the groups, while no difference was observed for the cognitive restraint subscale (*p* = 0.356). The PDSS total score also differed between the groups (*p* = 0.007) (Table 3).

The construct validity of the HRI-24 was evaluated using confirmatory factor analysis (CFA). The results indicated an acceptable model fit (χ^2^ = 485, df = 242, *p* < 0.001; RMSEA = 0.069; CFI = 0.874; TLI = 0.856), supporting the five-factor structure of the scale in the present sample. The standardized factor loadings ranged between 0.36 and 0.82, and all were statistically significant (*p* < 0.001). Measurement invariance analysis across the obesity and control groups supported configural invariance, followed by metric invariance (ΔCFI = −0.005) and acceptable scalar invariance, indicating that the scale structure was comparable between groups.

The BMI z-scores showed positive correlations with the HRI-24 total score (r = 0.166, *p* < 0.05) and the anthropophobia (r = 0.199, *p* < 0.01), agoraphobia (r = 0.218, *p* < 0.01), lethargy (r = 0.217, *p* < 0.01), and depressive mood (r = 0.146, *p* < 0.05) subscales, whereas no correlation was observed with the paranoia subscale (r = −0.037, *p* > 0.05). The BMI z-scores were also positively correlated with uncontrolled eating (r = 0.300, *p* < 0.001), cognitive restraint (r = 0.171, *p* < 0.05), and emotional eating (r = 0.268, *p* < 0.001). No significant correlation was detected between the BMI z-scores and the PDSS total score (r = 0.074, *p* > 0.05) (Table 4).

The PDSS total score showed positive correlations with the HRI-24 total score (r = 0.419, *p* < 0.001) and all HRI-24 subscales, including anthropophobia (r = 0.215, *p* < 0.01), agoraphobia (r = 0.144, *p* < 0.05), paranoia (r = 0.322, *p* < 0.001), lethargy (r = 0.494, *p* < 0.001), and depressive mood (r = 0.410, *p* < 0.001). In addition, the PDSS total score was positively correlated with uncontrolled eating (r = 0.400, *p* < 0.001) and emotional eating (r = 0.390, *p* < 0.001) and negatively correlated with cognitive restraint (r = −0.168, *p* < 0.05) (Table 4).

To further examine the independent contributions of psychosocial, behavioral, and sociodemographic factors, a multivariable binomial logistic regression analysis was conducted (Table 5). Among the psychosocial variables, emotional eating was independently associated with increased odds of obesity (OR = 2.25, 95% CI: 1.11–4.56, *p* = 0.024). In addition, the paranoia subscale was inversely associated with obesity (OR = 0.85, 95% CI: 0.76–0.95, *p* = 0.005). No significant independent associations were observed for the other HRI subscales, uncontrolled eating, cognitive restraint, or daytime sleepiness after adjustment. Among the sociodemographic variables, female gender was associated with lower odds of obesity (OR = 0.076, *p* < 0.001). Similarly, higher maternal education (OR = 0.233, *p* = 0.012) and higher paternal education (OR = 0.134, *p* = 0.001) were also associated with lower odds of obesity. Age and family income levels were not significantly associated with obesity status in the adjusted model.

## 4. Discussion

This study examined the relationships between obesity, social withdrawal characteristics, eating behaviors, and daytime sleepiness in adolescents. Given the cross-sectional design of the study, all findings should be interpreted as associations rather than causal relationships. The findings indicate that adolescents with obesity show higher levels of social withdrawal than their normal-weight peers, particularly in the domains of anthropophobia, agoraphobia, lethargy, and depressive mood, along with markedly increased tendencies toward uncontrolled and emotional eating. Although the daytime sleepiness levels were higher in the obesity group, no direct linear association was identified between BMI z-scores and daytime sleepiness. In contrast, the BMI z-scores were positively associated with dimensions of social withdrawal and eating behavior patterns, while daytime sleepiness demonstrated moderate associations with social withdrawal and eating behaviors. Taken together, these findings suggest that obesity in adolescence is not limited to disordered eating behaviors but is also associated with a broader spectrum of psychosocial and behavioral vulnerability, and obesity-related psychopathology may reflect a multidimensional structure.

These findings should be interpreted in light of the study’s initial research questions and hypotheses. First, in line with our expectations, adolescents with obesity were found to have higher levels of social withdrawal, dysregulated eating behaviors, and daytime sleepiness compared with their normal-weight peers. Second, the BMI z-scores were positively associated with multiple dimensions of social withdrawal and eating behaviors, consistent with the hypothesized relationships between weight status and these psychosocial factors. Third, although no direct association was observed between the BMI z-scores and daytime sleepiness, daytime sleepiness showed moderate associations with social withdrawal and eating behaviors, suggesting that it may function as an independent behavioral component within this biopsychosocial framework. Although several group differences reached statistical significance, the corresponding effect sizes were generally within the small-to-moderate range, indicating that these differences should be interpreted with caution regarding their clinical relevance.

Importantly, when all biopsychosocial variables were examined simultaneously in a multivariable model, only emotional eating remained independently associated with obesity status. This finding suggests that among the examined psychosocial and behavioral factors, emotional eating may represent a more proximal and clinically relevant mechanism linked to adolescent obesity. Notably, the paranoia subscale was inversely associated with obesity, indicating that certain dimensions of social withdrawal may relate differently to weight status. In contrast, the associations observed for other social withdrawal dimensions and daytime sleepiness in univariate analyses did not persist after adjustment. This pattern suggests that these factors may not independently predict obesity but rather operate through shared variance with other behavioral or emotional processes. These findings highlight the importance of distinguishing between correlational relationships and independent contributions within a biopsychosocial framework.

Our findings indicate that among adolescents with obesity, the distinguishing pattern is related not to the number of main meals but rather to the snacking frequency and particularly late-hour eating behaviors. While the number of main meals remained similar between groups, the obesity group reported a higher daily number of snacks and a higher prevalence of night eating. The current literature suggests that the association between snacking and obesity is often explained not merely by “how many snacks” are consumed but also by the energy density, content, and timing of snacks [29,30]. In particular, the consumption of energy-dense snacks late in the evening or close to sleep has been associated with a higher total energy intake and an increased risk of excess weight [31,32].

From a circadian nutrition perspective, later sleep timing and increased energy intake during the evening hours may adversely affect metabolic regulation [33,34]. Additionally, later bedtimes in adolescents have been associated with higher caloric intake after 8:00 PM and lower fruit and vegetable consumption; this pattern has also been linked to higher BMI [35,36]. In this context, the higher prevalence of night eating observed in the obesity group may reflect a dietary pattern in which energy intake shifts toward more irregular and circadian-misaligned time periods.

Moreover, the social and environmental context of snacking is also important. Snacking in conjunction with screen use and sedentary lifestyle patterns has been shown to be associated with poorer diet quality and increased obesity risk [37,38]. In our sample, adolescents with obesity reported spending more time at home and fewer social interactions, suggesting that snacking may occur in more sedentary and socially isolated contexts.

Finally, difficulties in eating control and a tendency toward late-hour “opportunistic eating” may represent behavioral mechanisms that negatively affect weight regulation [34]. Taken together, these findings suggest that intervention targets in adolescent obesity should move beyond focusing solely on meal frequency and instead address snack content, energy density, night eating behaviors, sleep timing, and the sedentary context in which eating occurs.

In this study, adolescents with obesity reported higher levels of social withdrawal risk, difficulties in eating control, and daytime sleepiness compared with the control group. The higher total scores on the Hikikomori Risk Inventory and particularly the anthropophobia, agoraphobia, and lethargy subscales in the obesity group suggest that obesity during adolescence may be associated with increased social avoidance tendencies and reduced behavioral activation. Recent studies indicate that there may be a bidirectional relationship between obesity and social withdrawal; weight-related stigma, difficulties in peer relationships, and internalizing symptoms may reinforce sedentary and home-centered lifestyle patterns [39,40]. The pronounced difference in the lethargy subscale, in particular, suggests that decreased motivation and lower behavioral engagement may be more prevalent among adolescents with obesity, potentially influencing physical activity levels indirectly.

From the perspective of eating behavior, the significantly higher levels of uncontrolled eating and emotional eating observed in the obesity group indicate that weight status is more strongly associated with impulsive and emotion-regulation-based eating patterns. In contrast, the absence of group differences in cognitive restraint suggests that adolescents with obesity may not develop deliberate compensatory restriction strategies to counteract overeating. The contemporary literature conceptualizes pediatric obesity not solely regarding excess energy intake but also through mechanisms such as increased reward sensitivity, poor emotion regulation, and eating as a coping strategy for stress [41,42]. Considering the association between emotional eating, depressive symptoms, and social withdrawal, negative affect may reinforce uncontrolled snacking and late-hour eating behaviors. The lack of differences in certain subscales, such as cognitive restraint and paranoia, may indicate that not all psychological or behavioral dimensions equally contribute to distinguishing adolescents with obesity from their normal-weight peers, highlighting the multidimensional and heterogeneous nature of obesity-related psychosocial profiles.

Furthermore, the higher levels of daytime sleepiness reported in the obesity group suggest that sleep dysregulation may accompany this behavioral pattern. Shortened sleep duration or circadian misalignment has been associated with alterations in appetite-regulating hormones, increased reward-driven eating, and impairments in executive functioning [43,44]. In adolescents, insufficient or irregular sleep may increase the consumption of energy-dense foods during evening hours and reduce inhibitory control [34,45]. In this context, daytime sleepiness may be considered a behavioral indicator of late bedtimes and night eating behaviors.

Taken together, these findings suggest that adolescent obesity is characterized not only by excessive caloric intake but also by interacting psychobehavioral factors such as social withdrawal tendencies, reduced behavioral activation, emotional and uncontrolled eating, and sleep dysregulation. Therefore, intervention programs should move beyond focusing solely on dietary restriction and instead incorporate multidimensional targets, including emotion regulation skills, sleep hygiene, enhancement of social participation, and strengthening of eating self-regulation.

The findings indicate that the BMI z-scores were significantly, albeit weakly to moderately, associated with social withdrawal risk and eating behavior characteristics. The positive correlations between the BMI z-scores and the anthropophobia, agoraphobia, lethargy, and depressive mood subscales suggest a gradual relationship between increasing weight status and higher tendencies toward social avoidance and reduced behavioral activation. In contrast, the absence of a significant association with the paranoia subscale suggests that obesity may be more closely related to internalizing and withdrawal-based dimensions rather than cognitive suspiciousness or paranoid ideation. The current literature reports significant associations between adolescent obesity, social isolation, internalizing symptoms, and reduced subjective well-being [46,47]. In this context, our findings suggest that as obesity increases, social avoidance tendencies may also intensify. Although these correlations reached statistical significance, their magnitude was generally small, indicating limited clinical effect and suggesting that these associations should be interpreted with caution.

From the perspective of eating behavior, the strongest associations of the BMI z-scores were observed with uncontrolled eating and emotional eating, supporting the view that weight gain is particularly linked to impulsive and emotion-driven eating patterns. This finding is consistent with contemporary models conceptualizing pediatric obesity regarding increased reward sensitivity, eating as a coping strategy for stress, and weakened inhibitory control [41,48]. Notably, a weak but significant positive correlation was also observed between the BMI z-scores and cognitive restraint. This may indicate that as the BMI increases, some adolescents attempt to exert cognitive efforts toward weight control; however, such efforts may be insufficient to counterbalance the tendencies toward uncontrolled and emotional eating. The literature suggests that cognitive restraint, particularly in adolescents, may paradoxically coexist with binge-eating tendencies and may not lead to sustainable long-term weight control [49].

On the other hand, the absence of a significant association between the BMI z-scores and daytime sleepiness suggests that sleep-related difficulties may be more closely linked to psychobehavioral factors rather than weight status per se. Indeed, the moderate positive correlations between daytime sleepiness and social withdrawal risk, particularly lethargy and depressive mood—indicate that sleep dysregulation is closely connected to social and emotional functioning. Furthermore, the moderate positive associations between daytime sleepiness and uncontrolled as well as emotional eating, along with the negative association with cognitive restraint, suggest that insufficient sleep may influence eating behavior through impairments in executive functioning and inhibitory control. Recent studies demonstrate that insufficient or irregular sleep increases reward-driven eating, weakens inhibitory control, and promotes a preference for energy-dense foods [44,50,51].

Taken together, this correlation pattern supports a biopsychosocial interaction model in adolescent obesity: Increased BMI appears to be associated with social withdrawal and emotional eating, whereas sleep dysregulation may function as an independent mediator or reinforcing factor linked to social withdrawal and impaired eating control, regardless of the weight level. These findings suggest that intervention programs should focus not only on weight reduction but also on improving sleep regulation, enhancing social participation, and strengthening emotion regulation skills.

Given the marked differences between groups regarding the gender distribution and parental education levels, the potential influence of confounding factors should be considered when interpreting the findings. In the present study, these variables were included as covariates in the multivariable regression model. After adjustment, key associations, particularly the role of emotional eating, remained significant. This suggests that the observed relationships are not solely attributable to demographic imbalances but reflect independent associations within the biopsychosocial framework. These findings may partly reflect gender-related differences in emotional and social functioning during adolescence.

The findings of this study should be interpreted in light of several methodological limitations. First, the cross-sectional design precludes causal inferences and does not allow determination of the directionality of the observed associations. The relationships between social withdrawal, eating behaviors, sleep characteristics, and obesity may be reciprocal and dynamic; however, the present design does not permit conclusions regarding temporal sequencing. Second, the sample exhibited a marked gender imbalance, with the majority of participants being female adolescents. Given that eating behaviors, body image concerns, social withdrawal, and sleep patterns may differ by sex during adolescence, the findings may more strongly reflect female adolescents and may have limited generalizability to males. Although multivariable regression analyses were performed to adjust for this imbalance, residual confounding cannot be entirely excluded. Third, the recruitment of the control group from the same clinical setting rather than from a community-based healthy population may have introduced selection bias and may limit the generalizability of the findings, as normal-weight adolescents presenting to a clinical setting may not fully represent the general population. Additionally, all psychological and behavioral measures were based on self-report instruments, which may be subject to recall bias and social desirability effects, particularly in the context of weight-related stigma. This reliance on self-report data may also increase the risk of common method bias, which could potentially inflate the observed associations, and eating behaviors assessed by the CTFEQ-17 may not fully capture culturally specific patterns. In addition, parental reports of income and educational level may be subject to reporting bias. The exclusion of participants who were unable to complete the assessment tools independently or who had incomplete or invalid questionnaire data may have introduced selection and attrition bias, particularly if non-completion was related to study variables such as social withdrawal, thereby limiting the generalizability of the findings. Although several correlations reached statistical significance, some effect sizes were small to moderate, reflecting the multifactorial nature of obesity and suggesting that no single psychobehavioral factor sufficiently explains the weight status. Given the number of statistical comparisons performed, there remains a potential risk of type I error, although appropriate correction methods were applied; therefore, the findings should be interpreted with caution. Finally, although sociodemographic and parental characteristics were collected, and multivariable modeling was applied to account for key sociodemographic variables, not all potential confounders could be fully included. Future longitudinal and multivariate studies are needed to more comprehensively test the proposed biopsychosocial framework and clarify the direction and mechanisms of these associations. The findings should therefore be interpreted with caution, as the cross-sectional nature of the study precludes conclusions regarding directionality or causality.

## 5. Conclusions

In conclusion, the present study highlights that adolescent obesity is associated not only with maladaptive eating behaviors but also with broader psychosocial vulnerabilities, including social withdrawal tendencies, reduced behavioral activation, emotional eating, and sleep-related difficulties. While the BMI was modestly associated with dimensions of social withdrawal and dysregulated eating, daytime sleepiness demonstrated moderate relationships with psychosocial and eating-related factors, suggesting that sleep disturbances may function as an important behavioral correlate independent of weight status. These findings support a biopsychosocial conceptualization of adolescent obesity, emphasizing the interplay between emotional regulation, social functioning, and sleep patterns alongside eating behaviors. Interventions targeting adolescent obesity may therefore benefit from moving beyond calorie-focused approaches and incorporating strategies aimed at improving emotion regulation, sleep hygiene, social engagement, and self-regulatory capacities. Future longitudinal research is warranted to clarify the directionality and potential mediating pathways between these interrelated factors. Notably, when biopsychosocial variables were examined in a multivariable model, only emotional eating remained independently associated with obesity status, highlighting its potential role as a key behavioral mechanism.

## Figures and Tables

**Table 1:** Sociodemographic and anthropometric characteristics of the groups.

Characteristics	Obesity Group (*n* = 100)	Control Group (*n* = 109)	Total (*n* = 209)
**Age (years), Mean ± SD**	15.4 ± 1.70	14.6 ± 1.72	15.0 ± 1.75
**Gender, *n* (%)**			
**-Female**	73 (73.0%)	104 (95.4%)	177 (84.7%)
**-Male**	27 (27.0%)	5 (4.6%)	32 (15.3%)
**Anthropometric Measurements**			
**-Weight (kg), Mean ± SD**	97.2 ± 18.2	56.3 ± 8.61	75.8 ± 24.8
**-Height (cm), Mean ± SD**	164 ± 8.54	167 ± 7.48	165 ± 8.66
**-BMI (kg/m^2^), Mean ± SD**	35.9 ± 4.73	20.3 ± 2.48	27.7 ± 8.07
**-BMI Z-Score (CDC), Mean ± SD**	2.27 ± 0.30	0.08 ± 0.76	1.13 ± 1.25
**Maternal Characteristics**			
**-Maternal Age (years), Mean ± SD**	42.8 ± 5.63	45.4 ± 4.80	44.2 ± 5.37
**-Maternal BMI (kg/m^2^), Mean ± SD**	30.7 ± 7.29	24.5 ± 3.99	27.5 ± 6.60
**-Maternal Education, *n* (%)**			
**--High school or below**	86 (86%)	34 (31.2%)	120 (57.4%)
**--University**	14 (14%)	75 (68.8%)	89 (42.6%)
**Paternal Characteristics**			
**-Paternal Age (years), Mean ± SD**	47.0 ± 7.33	48.5 ± 5.35	47.8 ± 6.40
**-Paternal BMI (kg/m^2^), Mean ± SD**	29.8 ± 4.90	27.3 ± 3.19	28.5 ± 4.30
**-Paternal Education, *n* (%)**			
**--High school or below**	89 (89%)	37 (33.9%)	126 (60.3%)
**--University**	11 (11%)	72 (66.1%)	83 (39.7%)
**Family Income Level, *n* (%)**			
**-Low**	14 (14.3%)	5 (4.6%)	19 (9.2%)
**-Middle**	48 (49.0%)	28 (25.7%)	76 (36.7%)
**-High**	36 (36.7%)	76 (69.7%)	112 (54.1%)

Notes: SD: standard deviation; BMI: body mass index; CDC: Centers for Disease Control and Prevention.

**Table 2:** Comparison of weight history, eating, and social habits of the groups.

Characteristics	Obesity Group (*n* = 100)	Control Group (*n* = 109)	Total (*n* = 209)	*p*-Value
**Eating Habits**				
**Weight Change, *n* (%)**				0.032
**-Increased**	52 (52.5%)	37 (33.9%)	89 (42.8%)	
**-Decreased**	23 (23.2%)	37 (33.9%)	60 (28.8%)	
**-No Change**	23 (23.2%)	35 (32.1%)	58 (27.9%)	
**Number of Meals, Mean ± SD**	2.72 ± 0.99	2.56 ± 0.70	2.64 ± 0.85	0.295
**Number of Snacks, Mean ± SD**	1.97 ± 1.10	1.60 ± 1.10	1.77 ± 1.11	0.005
**Skipping Meals, *n* (%)**				0.054
**-Yes**	24 (24.0%)	13 (11.9%)	37 (17.7%)	
**-No**	35 (35.0%)	50 (45.9%)	85 (40.7%)	
**-Sometimes**	41 (41.0%)	46 (42.2%)	87 (41.6%)	
**Night Eating, *n* (%)**				0.031
**-Yes**	44 (44.0%)	30 (27.5%)	74 (35.4%)	
**-No**	22 (22.0%)	37 (33.9%)	59 (28.2%)	
**-Sometimes**	34 (34.0%)	42 (38.5%)	76 (36.4%)	
**Social Habits**				
**Time Spent Indoors (hours/day), Mean ± SD**	4.17 ± 2.92	3.14 ± 2.36	3.63 ± 2.69	<0.001
**Social Relations (Yes), *n* (%)**	67 (67.0%)	101 (92.7%)	168 (80.4%)	<0.001

Note: SD: standard deviation.

**Table 3:** Comparison of scale scores between groups.

Scales/Subscales	Obesity Group (*n* = 100) Mean ± SD	Control Group (*n* = 109) Mean ± SD	t	*p*-Value	Cohen’s d
**Hikikomori Risk Inventory (HRI-24)**					
**-Total Score**	60.30 ± 16.77	53.17 ± 14.66	3.282	0.001	0.45
**-Anthropophobia**	9.14 ± 3.61	7.50 ± 2.76	3.717	<0.001	0.51
**-Agoraphobia**	8.33 ± 4.09	6.40 ± 2.63	4.087	<0.001	0.57
**-Paranoia**	19.56 ± 6.33	19.76 ± 5.42	0.248	0.805	0.03
**-Lethargy**	11.15 ± 4.07	8.83 ± 3.59	4.367	<0.001	0.60
**-Depressive Mood**	12.12 ± 4.17	10.67 ± 3.83	2.620	0.009	0.36
**Children Three-Factor Eating Questionnaire 17 (CTFEQ-17)**					
**-Uncontrolled Eating**	2.55 ± 0.64	2.06 ± 0.68	5.409	<0.001	0.75
**-Cognitive Restraint**	2.41 ± 0.83	2.30 ± 0.80	0.924	0.356	0.13
**-Emotional Eating**	2.21 ± 0.86	1.65 ± 0.77	5.029	<0.001	0.70
**Pediatric Daytime Sleepiness Scale (PDSS)**					
**-Total Score**	13.68 ± 6.14	11.48 ± 5.56	2.722	0.007	0.37

Notes: SD: standard deviation. Independent Samples *t*-test was used. Cohen’s d indicates effect size.

**Table 4:** Correlation matrix between BMI z-score and scale scores.

Variables	1	2	3	4	5	6	7	8	9	10	11
**1. BMI Z-Score**	—										
**2. HRI-24 Total**	0.166 *	—									
**3. Anthropophobia**	0.199 **	0.740 ***	—								
**4. Agoraphobia**	0.218 **	0.722 ***	0.621 ***	—							
**5. Paranoia**	−0.037	0.816 ***	0.453 ***	0.432 ***	—						
**6. Lethargy**	0.217 **	0.768 ***	0.447 ***	0.418 ***	0.510 ***	—					
**7. Depressive Mood**	0.146 *	0.797 ***	0.486 ***	0.451 ***	0.544 ***	0.596 ***	—				
**8. Uncontrolled Eating**	0.300 ***	0.308 ***	0.213 **	0.244 ***	0.136 *	0.368 ***	0.275 ***	—			
**9. Cognitive Restraint**	0.171 *	0.080	0.096	0.160 *	−00.017	−0.005	0.129	−0.069	—		
**10. Emotional Eating**	0.268 ***	0.394 ***	0.235 ***	0.212 **	0.277 ***	0.421 ***	0.374 ***	0.621 ***	0.015	—	
**11. PDSS Total**	0.074	0.419 ***	0.215 **	0.144 *	0.322 ***	0.494 ***	0.410 ***	0.400 ***	−0.168 *	0.390 ***	—

Notes: * *p* < 0.05, ** *p* < 0.01, *** *p* < 0.001. BMI: body mass index; HRI: Hikikomori Risk Inventory; PDSS: Pediatric Daytime Sleepiness Scale. The em-dash (—) indicates that the value is not applicable (diagonal elements).

**Table 5:** Multivariable binomial logistic regression predicting obesity status. Bold values indicate statistically significant results.

Predictor	β	OR	95% CI Lower	95% CI Upper	*p* Value
HRI-24 Subscales					
Anthropophobia	0.115	1.122	0.941	1.337	0.200
Agoraphobia	0.101	1.107	0.911	1.344	0.308
Paranoia	−0.160	0.852	0.762	0.953	**0.005** *
Lethargy	0.158	1.171	0.987	1.388	0.070
Depressive Mood	−0.056	0.946	0.803	1.113	0.500
CTFEQ Subscales					
Cognitive Restraint	0.070	1.073	0.578	1.990	0.824
Uncontrolled Eating	0.281	1.324	0.555	3.160	0.527
Emotional Eating	0.812	2.253	1.114	4.556	**0.024** *
PDSS					
Total PDSS Score	0.055	1.056	0.962	1.160	0.254
Sociodemographic Variables					
Age	0.177	1.194	0.904	1.576	0.212
Gender (Female vs. Male)	−2.574	0.076	0.017	0.350	**<0.001** *
Maternal Education (University vs. Lower)	−1.458	0.233	0.075	0.727	**0.012** *
Paternal Education (University vs. Lower)	−2.012	0.134	0.039	0.453	**0.001** *
Family Income (Level 1 vs. Level 0)	−0.490	0.613	0.107	3.519	0.583
Family Income (Level 2 vs. Level 0)	−1.190	0.304	0.051	1.829	0.194
Family Income (Level 3 vs. Level 0)	−1.728	0.178	0.028	1.120	0.066

Notes: β: unstandardized logistic regression coefficient; OR: odds ratio; CI: confidence interval; HRI-24: Hikikomori Rating Instrument-24; CTFEQ: Child Three-Factor Eating Questionnaire; PDSS: Pediatric Daytime Sleepiness Scale. Models estimated using a sample size of N = 207. * *p* < 0.05.

## Data Availability

The data presented in this study are available from the corresponding author upon reasonable request. Due to privacy and ethical restrictions, the data are not publicly available.
